# Potential Probiotic *Pediococcus pentosaceus* M41 Modulates Its Proteome Differentially for Tolerances Against Heat, Cold, Acid, and Bile Stresses

**DOI:** 10.3389/fmicb.2021.731410

**Published:** 2021-10-13

**Authors:** Mohd Affan Baig, Mark S. Turner, Shao-Quan Liu, Anas A. Al-Nabulsi, Nagendra P. Shah, Mutamed M. Ayyash

**Affiliations:** ^1^Department of Food Science, College of Agriculture and Veterinary Medicine, United Arab Emirates University, Al Ain, United Arab Emirates; ^2^School of Agriculture and Food Sciences, The University of Queensland, Brisbane, QLD, Australia; ^3^Department of Food Science and Technology, Faculty of Science, National University of Singapore, Singapore, Singapore; ^4^Department of Nutrition and Food Technology, Jordan University of Science and Technology, Irbid, Jordan; ^5^Food and Nutritional Science, School of Biological Sciences, The University of Hong Kong, Pokfulam, Hong Kong, SAR China

**Keywords:** environmental stress, proteomics, nano-LC-MS/MS, bacterial proteins, bile stress, acid stress

## Abstract

Probiotics containing functional food confer health benefits in addition to their nutritional properties. In this study, we have evaluated the differential proteomic responses of a potential novel probiotic *Pediococcus pentosaceus* M41 under heat, cold, acid, and bile stress conditions. We identified stress response proteins that could provide tolerances against these stresses and could be used as probiotic markers for evaluating stress tolerance. *Pediococcus pentosaceus* M41 was exposed for 2 h to each condition: 50°C (heat stress), 4°C (cold stress), pH 3.0 (acid stress) and 0.05% bile (bile stress). Proteomic analysis was carried out using 2D-IEF SDS PAGE and LC-MS/MS. Out of 60 identified proteins, 14 upregulated and 6 downregulated proteins were common among all the stress conditions. These proteins were involved in different biological functions such as translation-related proteins, carbohydrate metabolism (phosphoenolpyruvate phosphotransferase), histidine biosynthesis (imidazole glycerol phosphate synthase) and cell wall synthesis (tyrosine-protein kinase CapB). Proteins such as polysaccharide deacetylase, lactate oxidase, transcription repressor NrdR, dihydroxyacetone kinase were upregulated under three out of the four stress conditions. The differential expression of these proteins might be responsible for tolerance and protection of *P. pentosaceus* M41 against different stress conditions.

## Introduction

The gut microbiota of humans and animals plays key roles in regulation of nutrition, physiology, metabolism and immunity (Azad et al., 2018). Probiotics are known to provide protection against several diseases such as irritable bowel syndrome, diarrhea and gut inflammation, as well as maintaining the intestinal microflora and provide protection against gastric and gut pathogens such as *Helicobacter pylori* (Min et al., 2019). They causes reduction in lactose intolerance, exert protection against colon cancer, promote modulation of immune functions, increase calcium absorption and maintain blood cholesterol levels (Nagpal et al., 2012). Several mechanisms behind these beneficial properties of probiotics have been proposed but still need to be verified through molecular studies. Probiotic lactic acid bacteria (LAB) are important for food industry as they are commonly used for fermentation of food, beverages and dairy (Mbye et al., 2020). The selection of probiotics for their health benefits needs careful consideration through scientific evidence. Most of the probiotics are consumed from dairy based foods but non-dairy based probiotic foods have several benefits; they are suitable for vegetarians, provide protection from dairy based food allergens, provide less cholesterol to consumers of cardiovascular disease, enhance the nutritional properties of non-dairy foods, and prevent spoilage of meat based foods through pathogen inhibition (Min et al., 2019). Beneficial effects of probiotics and their counts can decrease due to several environmental stresses such as heat, cold, osmosis, high pressure, acid, and salt. These factors can induce oxidative stress in probiotics leading to formation of reactive oxygen species (ROS), changes in protein and metabolic functions which cause impairment of probiotic properties (Serrazanetti et al., 2009). Several studies reported adaptation of LAB to environmental stresses (De Angelis and Gobbetti, 2004; Muruzović et al., 2018; Mbye et al., 2020; Yang et al., 2021). Proteomic and transcriptomic techniques can reveal some complex regulatory networks in response to environmental stresses in bacteria.

In recent years LAB have received increased attention due to their adaptation and protective responses toward environmental stresses (Mbye et al., 2020; Yang et al., 2021). Many stress response proteins regulated by exposure of LAB to different stresses such as heat, cold, acid, bile and starvation have been studied using proteomic analysis (Alcántara and Zúñiga, 2012; Koponen et al., 2012; Bustos et al., 2015; Chen et al., 2017). However, no study has been carried out on proteomic response of *P. pentosaceus* under different stress conditions.

*Pediococcus* spp. is Gram-positive, non-motile, facultatively anaerobic, and non-spore-forming bacteria. Some strains of *P. pentosaceus* are probiotic LAB that are widely used for enhancing quality of fermented foods and pathogen inhibition (Kingcha et al., 2012; Jiang et al., 2021). A novel strain of *P. pentosaceus* M41 was isolated from a dried fish in our food microbiology laboratory and characterized as a potential probiotic (Alkalbani et al., 2019). The exopolysaccharide produced by *P. pentosaceus* M41 has remarkable physicochemical properties and health-promoting benefits (antitumor, antioxidative, antidiabetic, antibacterial) (Ayyash et al., 2020). The aim of this study was to explore proteomic responses of the potential probiotic *P. pentosaceus* M41 upon exposure to heat, cold, acid and bile stresses.

## Materials and Methods

### Bacterial Growth and Stress Treatment

*Pediococcus pentosaceus* M41 cultures stored at –80°C in 50% glycerol solution were revived in MRS broth (de Man Rogosa Sharpe broth, Lancashire, United Kingdom) and were incubated at 37°C for 20 h under anaerobic conditions according to Ayyash et al. (2020). Two consecutive subculturings in MRS broth were performed prior to stress treatments. Preliminary investigations were performed to determine the stress point of each stress factor. The pelleted culture was heated for 2 h at 40–65°C with 5°C interval/intervals. For cold stress, the pelleted culture was stored at 4°C for 1–5 h with 1 h intervals. The acid stress was determined by incubating the pelleted culture for 2 h under acid conditions pH 2.0 to 4.0 with 0.5 intervals. The bile stress was determined after the pelleted culture was subjected to different concentrations of a bile salt mixture (0.01–0.1%) (Sigma) for 2 h with 0.01% interval. The point achieved/achieved 1.0 log reduction or less was considered as stress point (data not shown).

**FIGURE 1:**
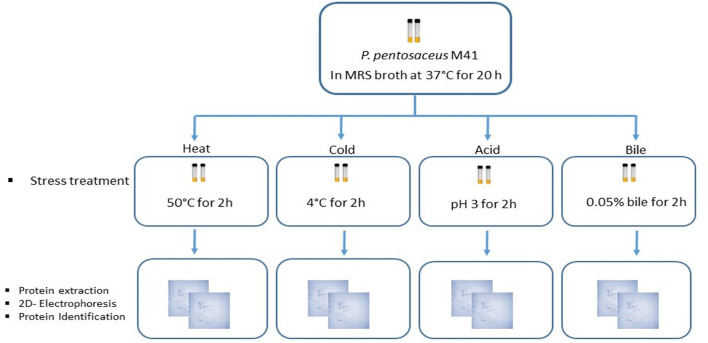
Workflow for the study of *P. pentosaceus* M41 under different stress treatments and its proteomic response for identification of differentially expressed proteins.

Two culture tubes per stress treatment were used (Figure 1). The *P. pentosaceus* M41 cultures in MRS broth in late exponential phase were subjected to heat stress for 2 h at 50°C. Cold stress treatment was performed by incubating the cultures at 4°C for 2 h. For acid and bile stress treatments, the cultures were centrifuged at 5000 × *g* for 10 min at 4°C and the cell pellets were resuspended in phosphate buffer pH 3.0 for acid stress and 0.05% bile salts for bile stress. The cultures were incubated at 37°C for 2 h. One set of culture tubes without stress were considered as the control. After stress treatments all the tubes were centrifuged at 5000 × *g* for 10 min at 4°C and the pellets were washed with phosphate buffer and stored at –20°C until protein extraction.

### Protein Extraction

Proteins were extracted from *P. pentosaceus* M41 cells exposed to different stress treatments using ReadyPrep^TM^ total protein extraction kit (Bio-Rad, United States) according to manufacturer instructions. The pellets were resuspended in 0.5 mL of 2-D sample buffer containing 7 M urea, 2 M thiourea, 40 mM Tris base, 1% *w*/*v* amidosulfobetaine-14 detergent and 0.001% bromophenol blue. The pellet suspension was transferred to a microcentrifuge tube and was placed on ice. The cells were lysed using an ultrasonic probe at 20 kHz (Sonifier SFX550 Model, Branson, CT, United States). The sonication was done four times using 30 s bursts. The pellet suspension was chilled on ice briefly between each ultrasonic treatment. The tubes were centrifuges at 10,000 × *g* for 20 min. The supernatant containing bacterial proteins was transferred to a clean microcentrifuge tube and protein concentration was estimated using the Bradford assay (Bradford, 1976).

### Two-Dimensional Isoelectric Focusing Sodium Dodecyl Sulfate Polyacrylamide Gel Electrophoresis

For isoelectric focusing (IEF), 400 μg of bacterial proteins in a total volume of 300 μL in sample buffer was loaded onto 17 cm, pH 4.0–7.0, non-linear, IPG strips (Ready Strip^TM^, Bio-Rad, United States) in a rehydration tray. Two mL of mineral oil was applied to each strip and kept overnight at 20 °C for passive rehydration of proteins. The strips were then transferred to an IEF tray (Protean II IEF^TM^ cell, Bio-Rad, United States) and protein focusing was performed at 20°C with a total 65,000 volt hour (Vh) current supply. Following IEF, the strips were applied with reduction and alkylation buffer for 20 min each and then rinsed with ultrapure water. For second dimension SDS PAGE, the strips were placed on 12% polyacrylamide gels and electrophoresis was performed in Protean II XL Cell^TM^ (Bio-Rad, United States) with a total current flow of 200 V at 10°C. The gels were run in duplicate to ensure reproducibility. After second dimension electrophoretic run, the gels were stained with Coomassie blue for protein visualization. The gels were scanned with a gel documentation system (Gel Doc^TM^, Bio-Rad, United States). The protein spots from all the stress treatments and the control were compared for differential expression analysis using Melanie^TM^ Version 9.2.3 (Swiss Institute of Bioinformatics, Lausanne, Switzerland). The protein spot intensities having more than 1.5 fold and lower than 0.5 fold change were considered as upregulated and downregulated respectively as compared to control. The selected protein spots were picked in 0.2 mL PCR tubes and 100 μL of ultrapure water was added and stored at 4°C.

### Enzymatic Digestion and Protein Identification

The protein identification process was performed by BGI Genomics (Beijing, China). The dye from the spots was removed by washing with 1mL of a decoloring solution (50% acetonitrile + 25 mM ammonium bicarbonate solution) several times until the gel spot was decolorized. After cleaning, 500 μl of acetonitrile was added to gel spots for dehydration. The supernatant was removed and the gel spot was dried in air. The gel spots were added with 10 mM DTT (dithiothreitol) at 56°C for 1 h to reduce the disulfide bond. The supernatant was removed and 55 mM IAM (iodoacetamide) was added in a dark room for 45 min to perform alkylation of cysteine. The supernatant was removed and the gel spots were dried in air. An aliquot of 0.1 μg/μl trypsin solution was added and the tubes were kept on ice for 30 min, and 25 mM ammonium bicarbonate solution was added to cover the gel spots and digested overnight at 37°C. The remaining liquid outside the gel spot was transferred to a new centrifuge tube. The peptide was extracted once from the gel spot with 50% acetonitrile-water and again with 100% acetonitrile. The two extracted solutions were combined with the tryptic digested solution and lyophilized.

### Phase Nano-Liquid Chromatography-Mass Spectrometry Analysis

The lyophilized peptide samples were reconstituted with mobile phase A (2% ACN, 0.1% FA), shaken and centrifuged, and the supernatant was taken. The samples were separated on a Shimadzu LC-20AD Nano nanoliter liquid chromatograph. The sample was first loaded to a trap column for enrichment, and then the sample was eluted with a sharp gradient at a flow rate of 300 nl/min with a self-packed C18 column (75 μm × 15 cm, 3 μm, 120 Å). The nanoliter liquid chromatograph end was directly connected to a mass spectrometer LTQ Orbitrap Velos (Thermo Fisher, United States), and the ion source was nanoESI. In the data acquisition, the positive ion scanning mode was adopted, the resolution of the MS1 was 30,000, and the scanning mass range was 350–1,500 m/z. Based on the MS1 scanning information, according to the ion intensity in the order MS1 from high to low, the top six were selected for fragmentation and the MS2 information was scanned in Orbitrap analyzer. The resolution of the MS2 was 7,500, and the fragmentation mode was HCD.

### Statistical Analysis

The spot intensities were quantitatively detected by Melanie^TM^ Version 9.2.3 (Swiss Institute of Bioinformatics, Lausanne, Switzerland). For differentially expressed proteins the spot intensities were statistically analyzed by using Metaboanalyst 5.0 software (Chong et al., 2018). The spot intensities were log2 transformed for correlation analysis, principal component analysis (PCA), partial least squares-discriminant analysis (PLS-DA) and heatmap. The analysis was performed on two biological replicates using one-way analysis of variance (ANOVA). All the values are presented as the mean ± standard error (SE).

## Results and Discussion

### Identification of Differentially Expressed Proteins in *Pediococcus pentosaceus* M41 and Their Functional Categorization

**FIGURE 2:**
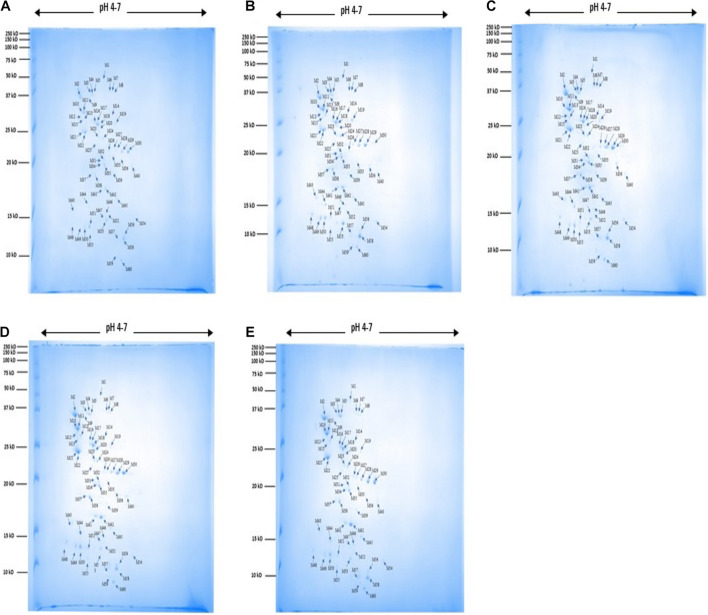
2DE gel images for protein expression profile of *P. pentosaceus* M41 under heat, cold, acid and bile stresses. **(A)** Control, **(B)** heat, **(C)** cold, **(D)** acid, **(E)** bile. The arrow indicates proteins which were quantitatively analyzed by Melanie^TM^ Version 9.2.3 (Swiss Institute of Bioinformatics, Lausanne, Switzerland). The protein spots were identified by PhaseNano-LC-MS/MS analysis and assigned identities/functions are listed in Table 1.

A total of 60 protein spots were quantitatively detected under control, heat, cold, acid, and bile stress treatments (Figures 2A–E), out of which 29 proteins under acid stress (21 upregulated and 8 downregulated), 33 proteins under bile stress (25 upregulated and 8 downregulated), 31 proteins under heat stress (28 upregulated and 3 downregulated) and 21proteins under cold stress (18 upregulated and 3 downregulated) were differentially expressed compared to the control. Seven upregulated proteins were common among all the stress treatments which were spot M12, spot M25, spot M31, spot M40, spot M41, spot M43, and spot M53. One downregulated protein (spot M22) was common among all the stress treatments (Table 1).

**TABLE 1:** Identified proteins in *P. pentosaceus* M41 with their molecular weight, *pI* values, protein spot intensities under heat, cold, acid, and bile stresses.

Function	Homologous protein	Molecular weight (kDa)	pI	Spot no.	Induction factor (intensity)			
					Heat	Cold	Acid	Bile
Stress response	Chaperone protein DnaK	64685.24	4.31	M2	41.2	31.0	34.7	36.8
	ATP-dependent Clp protease ATP-binding subunit	78048.17	4.59	M6	9.5	17.0	18.5	16.9
	60 kDa chaperonin	57395.94	4.43	M11	34.8	41.0	37.0	20.6
	DNA protection during starvation protein	18684.00	5.65	M30	140.5	86.0	148.2	76.6
	Protein GrpE	22832.00	4.56	M59	15.8	18.0	24.3	7.36
Carbohydrate metabolism	PTS transporter subunit EIIC	70656.40	7.70	M8	12.6	7.0	15.0	24.3
	Phosphoenolpyruvate-protein phosphotransferase	62607.52	4.28	M9	33.8	32.0	42.8	28.7
	ATP-dependent 6-phosphofructokinase	35752.50	5.64	M40	25.3	29.0	40.5	30.9
	Fructose-bisphosphate aldolase	32197.28	4.81	M41	47.5	33.0	57.9	67.7
	Mannose permease IID component	30908.26	9.34	M45	48.6	35.0	34.7	36.8
	Enolase	46382.00	4.96	M23	31.7	25.0	27.7	25
	Polysaccharide deacetylase family protein	24076.34	5.57	M24	69.7	53.0	55.5	36.8
Amino acid metabolism	Argininosuccinate lyase	52050.00	4.56	M4	40.1	43.0	67.1	38.3
	IGP synthase cyclase subunit	25129.60	4.19	M25	27.4	18.0	23.1	40.5
	Indole-3-glycerol phosphate synthase	28219.00	6.03	M33	27.4	15.0	11.5	27.2
Protein metabolism	HTH-type transcriptional regulator	27470.82	9.73	M7	14.7	9.0	13.8	21.3
	Ribosomal RNA small subunit methyltransferase	28062.00	4.43	M10	41.2	32.0	25.4	15.5
	Ribosome maturation factor RimM	21289.00	4.68	M14	33.8	15.0	25.4	34.6
	Elongation factor Tu	43232.01	4.60	M18	124.7	114.0	37.0	105.0
	50S ribosomal protein L2	29956.00	5.65	M31	24.3	19.0	19.6	24.3
	Transcriptional repressor NrdR	18702.00	5.65	M32	51.7	45.0	25.4	58.2
	Elongation factor Ts	36694.98	4.63	M35	98.2	112.0	72.9	76.6
	30S ribosomal protein S2	28729.96	5.11	M38	133.1	126.0	140.1	124.0
	Bifunctional protein PyrR	19712.58	4.78	M47	33.8	18.0	13.8	27.2
	Ribosomal RNA large subunit methyltransferase	17750.00	6.03	M52	106.7	59.0	16.2	26.5
	30S ribosomal protein S18	8954.00	6.01	M56	16.9	31.0	22.0	62.6
	50S ribosomal protein L10	17543.44	4.76	M60	62.3	62.0	67.1	15.5
Nucleotide metabolism	ATP synthase subunit beta	50620.02	4.30	M15	51.7	37.0	24.3	46.4
	Primosomal protein DnaI	33401.10	6.01	M22	12.6	12.0	6.9	31.7
	Ribose ABC transporter (ATP-binding protein)	54006.28	5.11	M21	84.5	71.0	55.5	54.5
	Uncharacterized ABC transporter ATP-binding protein	34312.00	5.65	M29	67.6	82.0	77.5	71.4
	Adenylate kinase	23661.13	4.75	M46	17.9	7.0	26.6	30.9
	Exodeoxyribonuclease 7 small subunit	8846.00	4.56	M54	30.6	9.0	18.5	6.63
	Putative ABC transporter ATP-binding protein YheS	71530.75	4.69	M55	21.1	52.0	22.0	14.0
Cell wall synthesis	UDP-*N*-acetylmuramate–L-alanine ligase	59868.00	4.60	M17	47.5	36.0	75.2	64.0
	Putative tyrosine-protein kinase CapB	25217.00	5.65	M36	25.3	29.0	40.5	30.9
Oxidoreductases	Lactate oxidase	38810.21	5.13	M26	31.7	19.0	16.2	31.7
	4-Hydroxymandelate oxidase	39738.36	4.79	M34	28.5	19.0	20.8	48.6
	Aldo/keto reductase	32618.57	5.22	M39	38.0	40.0	28.9	44.2
Miscellaneous proteins	3-Methyl-2-oxobutanoate hydroxymethyltransferase	31123.00	9.73	M1	8.4	9.0	37.0	41.2
	Molybdopterin/thiamine biosynthesis adenylyltransferase	37696.98	4.56	M43	48.6	26.0	25.4	64.8
	Cell division protein FtsZ	44008.19	4.27	M13	64.4	31.0	26.6	25.8
	UPF0210 protein FVP42_08325	46539.45	4.96	M20	38.0	14.0	17.3	32.4
	Lactate 2-monooxygenase	44207.00	5.09	M27	17.9	13.0	10.4	27.2
	Probable dihydroxyacetone kinase regulator	23756.08	6.03	M37	42.2	47.0	30.1	47.1
	Uncharacterized protein	33626.86	4.29	M50	89.8	86.0	86.8	69.2
	Uncharacterized hydrolase YsaA	29509.21	4.39	M53	21.1	15.0	39.3	67.7
	Uncharacterized protein	19021.69	7.18	M58	146.9	93.0	69.4	103.0
	Uncharacterized protein	24566.92	6.55	M3	26.4	22.0	30.1	22.8

*The proteins were categorized according to their biological functions.*

**FIGURE 3:**
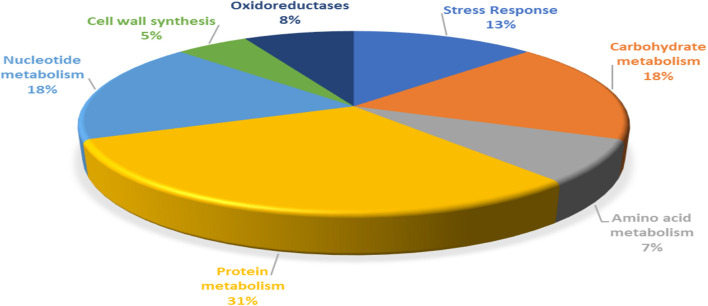
Functional categorization of *P. pentosaceus* M41 proteins identified under heat, cold, acid, and bile stresses.

For functional categorization, the identified protein ID’s were searched against UniProt database and the proteins were assigned to different cellular functions (Figure 3). Maximum 31% of identified proteins were found to be associated with protein metabolism followed by 18% of carbohydrate and nucleotide metabolism each. In addition, 13% proteins were found to be associated with stress responses, 8% proteins were involved in oxidation reduction, 7% proteins with functions in amino acid metabolism and 5% proteins were involved in cell wall synthesis.

### Multivariate Data Analysis

**FIGURE 4:**
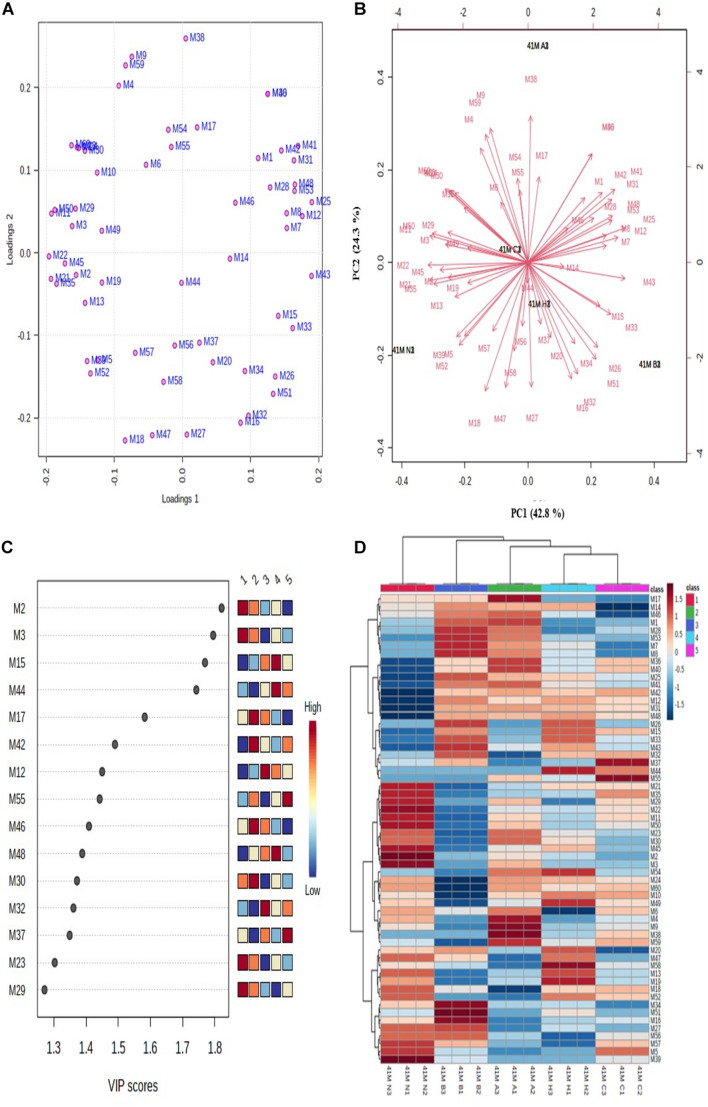
Multivariate data analysis using MetaboAnalyst software. **(A)** PCA loading plot; **(B)** PCA biplot for variations among differentially expressed proteins, **(C)** VIP score plot for proteins with maximum abundance, and **(D)** hierarchical clustering and correlation heatmap. Each colored cell in heatmap represents average peak intensity value in Table 1. The column represents stress treatments and rows represents proteins (41 N = control, 41 A = acid, 41 B = bile, 41 C = cold, and 41 H = heat). The color red indicates increased and blue indicates decreased *Z* scores which represent standard deviation from the mean as compared to the control.

For analyzing correlation between protein profiles and changes between control, heat, cold, acid and bile treatments, multivariate data analysis was performed by using MetaboAnalyst software (Figures 4A–D). Before analysis, the differences in spot intensities for all the proteins were normalized by log transformation.

#### Principal Component Analysis

The unsupervised PCA biplot was created from loadings plot which represents the influence of different stresses on principal component (PC) (Figures 4A,B). The PCA score represents the variation in protein profile due to the influence of different stress treatments. The PCA plots showed clear separation of proteins among different PCs which represents the differential expressions of proteins. The first principal component (PC1) showed 42.8% variation among all the protein intensities whereas the second principal component (PC2) showed 24.3% variation. The differentially expressed proteins with the maximum abundance were identified with a variable importance in projection (VIP) plot with VIP scores ranging from 1.3 to 1.8 (Figure 4C).

#### Hierarchical Clustering and Correlation Heatmap

The relative changes between differentially expressed proteins under different stress treatments were observed by hierarchical clustering and correlation heatmap (Figure 4D). Chaperone protein DnaK (spot M2), uncharacterized protein (spot M3), ATP synthase subunit beta (spot M15), UDP-*N*-acetylmuramate–L-alanine ligase (spot M17), 60 kDa chaperonin (spot M12), putative ABC transporter ATP-binding protein YheS (spot M55), adenylate kinase (spot M46), DNA protection during starvation protein (M30), transcriptional repressor NrdR (spot M32), probable dihydroxyacetone kinase regulator (spot M37), enolase (spot M23) and uncharacterized ABC transporter ATP-binding protein (spot M29) were the most abundant proteins identified.

### Differential Proteomic Responses of *Pediococcus pentosaceus* M41 Under Heat, Cold, Acid, and Bile Stresses

The exposure of LAB to different environmental stresses could be a great challenge for ever growing food industry. Different stresses such as high or low temperature, low pH, osmotic pressure due to food processing and gut environment can impair or reduce the biological properties of LAB. Several studies reported adaptation of LAB to different environmental stresses which could be due to underlying changes in gene transcription and protein expression (De Angelis and Gobbetti, 2004).

In the food industry, the processing of food products requires heating at different temperatures. Therefore, the LAB are frequently exposed to heat stress in LAB fortified foods. Several probiotic bacteria were studied by other researchers under heat stress conditions. LAB which can survive high temperatures were then identified. The mechanism of adaptation of LAB under stress conditions involves upregulation of different stress responsive proteins. *Lactobacillus kefiranofaciens* M1 adapts itself under stress conditions by increasing the expression of heat shock proteins (HSPs), enzymes such as chaperone protein DnaK prolyl-tRNA synthase, phosphoenolpyruvate-protein phosphotransferase, cofactors (GroES) and chaperonins (GroEL) (Hernández-Alcántara et al., 2018). These proteins promote proper folding and translocation of newly synthesized polypeptides (Hernández-Alcántara et al., 2018). Different proteins were identified in LAB exposed to heat stress such as DnaK, GroEL, and GroES in *Lb. plantarum* DPC2739 and DPC2741 (De Angelis and Gobbetti, 2004), enolase, and glyceraldehyde-3-phosphate dehydrogenase in *Lb. helveticus* PR4 (Di Cagno et al., 2006), small heat shock proteins (HSP1 and HSP3) and chaperonins (DnaK, GrpE, GroEL, and GroES) in *Lb. plantarum* (Lp 813 and Lp 998) and *Lb. casei* (ATCC393) (Haddaji et al., 2015; Ferrando et al., 2016), small heat shock proteins (HSP27 and HSP72) in *Lb. rhamnosus* GG (ATCC 53103), *Lb. johnsonii* P47-HY, and *Lb. reuteri* P43-HUV (Liu et al., 2015).

Dairy-based food products such as cheese, yogurt and ice cream require storage at low temperatures which expose LAB to cold stress. Some LAB can survive at low temperatures which is important for their commercial use because of their storage and transport at chilled to subzero temperatures (Fonseca et al., 2015; Zhang et al., 2016). Cold stress can alter the protein expression in LAB. Cold-shock and anti-freeze proteins protect LAB under low temperature stress. Cold-stress induced proteins were identified in LAB such as cold-shock proteins (cspC, cspL, and cspP) and ATPase in *Lb. plantarum* L67 (Song et al., 2014). Enzymes that activate membrane fluidity and membrane phospholipids were identified in *Lb. bulgaricus* (ATCC 11842 and CFL1) (Meneghel et al., 2017). Anti-freeze and ice nucleation proteins were found in *Lb. paracasei* and *Lb. mali* (Polo et al., 2017).

During intestinal digestion LAB are exposed to acidic pH which can affect their function and survival. Food products with acidic pH such as dairy-based fermented foods and pickles can also exposes LAB to acid stress. Some LAB can survive under acidic environments due to the presence of molecules such as proton-translocating ATPase which protect the LAB by stabilizing the intracellular pH (Šeme et al., 2015; Pérez Montoro et al., 2018; Wang et al., 2018). Some acid stress induced proteins identified in LAB include glyceraldehyde-3-phosphate dehydrogenase and DnaK in *Lb. casei* Zhang (Wu et al., 2011); GrpE, methionine synthase, and 30S ribosomal protein S2 in *Lb. plantarum* LC 804, CIP A159, and CECT 4185 (Hamon et al., 2011); 2,3-bisphosphoglycerate-dependent phosphoglycerate mutase 2 (PGAM-d); elongation factor G, and DnaK in *Lb. pentosus* AP2-15, AP2-18, and LP-1 (Pérez Montoro et al., 2018). Proteins related to purine biosynthesis, galactose metabolism, and fatty acid biosynthesis were also identified in *Lb. rhamnosus* GG (ATCC 53103) (Koskenniemi et al., 2009).

The probiotic action of LAB depends on its ability to survive under gastrointestinal conditions where they are exposed to acidic environments and bile salts. Bile salts can cause DNA damage as well as changes in protein conformation and oxidative stress (Merritt and Donaldson, 2009). Several studies reported the responses of LAB under bile stress. Induction of stress responsive proteins was observed in different LAB species when exposed to bile stress (Alcántara and Zúñiga, 2012). Proteins identified in LAB upon exposure to bile stress were glutathione reductases and cyclopropane fatty acyl phospholipid synthase in *Lb. plantarum* 299 V, LC 804, and LC 56 (Hamon et al., 2011), in addition to bile salt hydrolases and F0F1-ATPase (+) in *Lactobacillus* and *Bifidobacterium* (Ruiz et al., 2013).

The extensive applications of LAB in the food industry make it imperative to link their important characteristics with their corresponding genes and proteins (Sharma et al., 2020). Stress responsive genes and proteins could be helpful in marker-assisted selection of LAB which are more resistant to adverse environments.

In our study, *P. pentosaceus* M41 responded to different stresses by maximum increases in expression of proteins related to protein and nucleotide metabolism which indicate the tolerance of *P. pentosaceus* M41 to these stresses through growth and repair mechanisms. The proteome of *P. pentosaceus* M41 under heat, cold, acid and bile stresses showed several proteins involved in stress response and tolerance. The maximum number of proteins identified was involved in protein metabolism which mainly regulates transcription and translation such as ribosomal RNA small subunit methyltransferase, ribosome maturation factor RimM, elongation factor Tu, 50S ribosomal protein L2, elongation factor Ts, 30S ribosomal protein S2, ribosomal RNA large subunit methyltransferase, 30S ribosomal protein S18 and 50S ribosomal protein L10. The ribosomal proteins regulate RNA synthesis and proteins involved in ribosome assembly. They are ubiquitous proteins primarily involved in extra-ribosomal functions (Warner and McIntosh, 2009). 50S ribosomal protein L2 (spot M31) and 30S ribosomal protein S2 (spot M38) were upregulated under all the stress treatments.

#### Stress Responsive Proteins

The identified proteins which were involved in stress responses are chaperone protein DnaK, ATP-dependent Clp protease, ATP-binding subunit, 60 kDa chaperonin, DNA protection during starvation protein and protein GrpE. Chaperones are important proteins which regulate cell homeostasis and stress responses (Ranford et al., 2000). Chaperone protein DnaK is an ATP dependent major chaperone which maintains protein homeostasis and functions along with other co-chaperones, a J-domain protein (JDP) and a nucleotide exchange factor. AtcJ is a short JDP co-chaperone which was identified in *Shewanella oneidensis* and is responsible for cold adaptation (Maillot et al., 2019). A group of molecular chaperones comprise of chaperonins which are defined by their sequence similarity. Chaperonins with 60 kDa monomers have 14 subunits arranged in two rings which form large protein complexes. They cause protein folding in a protected environment in which the unfolded proteins cannot interact with other proteins (Lund, 2009). Chaperone protein DnaK negatively regulates heat shock responses and causes decreases in heat shock proteins (HSP) in *Caulobacter crescentus* (Da Silva et al., 2003). Sarbeng et al. (2015) reported that DnaK-ATP dimer is important for interaction with Hsp40 heat shock protein which is responsible for its functioning and maintenance of proteostasis. In our study downregulation of chaperone proteins under all the stress treatments indicates possible activation of HSP’s for tolerance against stress conditions. ATP-dependent Clp protease (spot M6) is a serine peptidase which regulates homeostasis, stress response, cell differentiation and virulence in bacteria. Clp alone and in conjunction with Hsp100 (Clp-ATPase) cause protein degradation. It was reported that deletion of *clpP* gene in *B. subtilis* reduced heat tolerance and survival (Malik and Brötz-Oesterhelt, 2017). In our study this enzyme was upregulated under acid and cold stresses while being downregulated under heat stress. The HTH-type transcriptional regulator (spot M7) belongs to MerR family of transcription regulators which helps survival of bacteria under different stress conditions by its C-terminal binding domain that activates transcription of stress response genes (Singh et al., 2018). The upregulation of the HTH-type transcriptional regulator under acid, bile and heat stresses indicates possible activation of stress response genes which might provide tolerance to *P. pentosaceus* M41.

#### Carbohydrate Metabolizing Proteins

Carbohydrates are important sources of energy for bacterial cell metabolism. Phosphoenolpyruvate-phosphotransferase system (PEP-PTS) (spot M9) regulates phosphorylation and transport of selected carbohydrates in response to their availability and maintains many cellular functions (Houot et al., 2010). Bacteria utilize carbohydrate sources for efficient signaling network. The enzyme IIC (EIIC) (spot M8) as part of the PEP-PTS regulates transport of sugar molecules across inner bacterial membrane. The phosphorylation of sugar prevents its efflux across the membrane (McCoy et al., 2015) and conserves ATP. The enzyme PTS was upregulated in *P. pentosaceus* M41 under all the stress treatments while EIIC was upregulated in acid, bile, and heat stresses. The upregulation of these carbohydrate metabolizing enzymes might protect *P. pentosaceus* M41 from different stresses by activation of stress signaling.

Polysaccharide deacetylases (spot M24) are carbohydrate metabolizing enzymes which catalyze metal-dependent deacetylation of *O*- or *N*-acetylated bacterial cell wall polysaccharides. The carbohydrate metabolizing enzymes of LAB serve as potential probiotic markers which indicate adaptation of these bacteria to the human GI tract (Abriouel et al., 2017; Andreou et al., 2018). This enzyme was upregulated under heat, cold and acid stresses and it could be used as probiotic markers in *P. pentosaceus* M41 as well.

#### pH Homeostasis-Related Proteins

Proper functioning of proteins depends on many external factors such as heat, cold, osmotic pressure, pH, ionic strength, toxins, heavy metals etc. Probiotics are subjected to several types of stresses during oral, gastric and intestinal digestion. 3-Methyl-2-oxobutanoate hydroxymethyltransferase (spot M1) is involved in the biosynthesis of pantothenic acid which is essential for energy metabolism in all organisms including bacteria. Pantothenic acid regulates activities of various enzymes and is involved in protein and lipid metabolism and promotes growth in *Lactobacillus helveticus* (Yao et al., 2018). This protein was upregulated under acid and bile stresses which might provide tolerance to *P. pentosaceus* M41against these stresses. Changes in pH and exposure to bile salts are two prominent stresses which can affect probiotic function. Cell division protein FtsZ (spot M13) is important for cytokinesis in bacteria. FtsZ forms protofilaments in the presence of GTP at pH 7.0. Santra and Panda (2007) reported loss of GTPase activity of FtsZ which caused formation of aggregates instead of protofilaments when exposed to pH 2.5. Interestingly the functional state of FtsZ was recovered upon exposure to pH 7.0 (Santra and Panda, 2007). Protein FtsZ was downregulated in acid and bile stresses which is in accordance with a previous study (Santra and Panda, 2007). However, FtsZ was upregulated under heat stress.

#### Cell Wall Proteins

UDP-*N*-acetylmuramate–L-alanine ligase (spot M17) is an important cell wall synthesizing enzyme in both Gram positive and Gram-negative bacteria. Feng et al. (2019) reported an overexpression of cell wall synthesizing enzymes in heat and acid tolerant *Alicyclobacillus acidoterrestris* when exposed to 65°C. The upregulation of UDP-*N*-acetylmuramate–L-alanine ligase in heat, acid and bile stresses might be responsible for tolerance of *P. pentosaceus* M41 against these stresses which is consistent with study of Feng et al. (2019).

Elongation factor Tu (spot M18) is a universally conserved multifunctional protein present on the surface of both Gram positive and Gram negative bacteria. It functions as GTPase that maintains translational accuracy. Ef–Tu interacts with several other proteins and regulates different cellular processes (Widjaja et al., 2017). The mucus layer of human intestinal epithelial cells is an attachment site for LAB. Proteins containing a mucus binding domain (MUB) were identified in *Pediococcus pentosaceus* and other LAB species. MUB assists in attachment of LAB to the mucus layer (Boekhorst et al., 2006). Ef-Tu promotes pH dependent binding of *Lactobacillus reuteri* and *Lactobacillus johnsonii* to GI mucosal surfaces by binding with glycolipids and sulfomucin through sulfated carbohydrate moieties (Granato et al., 2004; Nishiyama et al., 2013). Ef-Tu was downregulated under acid, bile and cold stresses which might prevent *P. pentosaceus* M41 from binding to the mucosal surface under these stresses.

Lactic acid is a primary metabolite secreted by LAB and reduces the pH of their surroundings to about 4 or below. The cell surface proteins play an important role in attachment of LAB to intestinal mucosa. Enolase (spot M23) and glyceraldehyde-3-phosphate dehydrogenase are glycolytic enzymes present on the cell surface of many Gram-positive bacteria at an acidic pH and are released into the environment at an alkaline pH. Lipoteichoic acids mediate binding of these proteins to the cell surface at a pH below their isoelectric point. Lactobacilli rapidly modify their surface properties in response to changes in pH (Antikainen et al., 2007). Enolase was upregulated in *P. pentosaceus* M41 under heat and acid stresses which might help this bacterium in binding to the cell surface under stress conditions.

#### Amino Acid Metabolizing Proteins

The heterodimeric enzyme imidazole glycerol phosphate (IGP) synthase (spot M25) catalyzes histidine biosynthesis which plays an important role in cellular metabolism by interconnecting nitrogen metabolism and purine synthesis (Chioccioli et al., 2020). This enzyme was upregulated under all the stress treatments and could be responsible for providing tolerance to *P. pentosaceus* M41 through growth and repair by increased histidine biosynthesis.

Lactate oxidase (spot M26) catalyzes conversion of lactate to pyruvate. The aerobic reaction utilizes molecular oxygen for oxidation of lactic acid which increases intracellular concentration of pyruvate. Barré et al. (2007) reported an overexpression of lactate oxidase in *Lactococcus lactis* exposed to copper stress. This enzyme was overexpressed under heat, cold and bile stresses (Barré et al., 2007).

#### Nucleotide Metabolizing Proteins

DNA protection starvation protein (Dps) is a DNA-binding protein from starved cells, which provides protection to cells exposed to severe environmental stresses such as nutritional deprivation and oxidative stress. Dps were found to protect *E. coli* from different stresses which include thermal stress, metal stress, oxidative stress, radiations (UV and gamma), and high osmotic pressure. The mechanism of action of Dps for protection against various stresses is due to its three functional properties which are ferroxidase activity, DNA binding and iron sequestration. The multifaceted action of Dps is important in protection against acid stress, iron and hydrogen peroxide detoxification (Jeong et al., 2008; Calhoun and Kwon, 2011). In our study *P. pentosaceus* M41 showed upregulation of Dps (spot M30) under acid and heat stresses which could provide tolerance against these stresses and downregulated under bile and cold stresses.

The bacterial DNA replication involve several genes such as *dnaA*, *dnaB*, *dnaI*, and *polA*, primosome assembly genes and ribonucleotide reductases (RNR) genes (Rodionov and Gelfand, 2005). The transcription repressor NrdR (spot M32) is usually clustered with these genes. NrdR along with Fur regulates expression of RNRs and interacts with thioredoxin (TrxA) which has an important regulatory role in redox homeostasis and signal transduction in bacteria (Naveen and Hsiao, 2016). The NrdR transcription repressor was upregulated in *P. pentosaceus* M41 under heat, cold and bile stresses while being downregulated under acid stress.

The molecular mechanisms underlying bacterial responses to changing environments include alterations in gene expression and post translational modifications of proteins. Phosphorylation and dephosphorylation of proteins is important for their proper functioning and regulation of cellular and molecular functions in living organisms. In bacteria the phosphotransferase PTS system causes phosphorylation of proteins at histidine and aspartic acid which in turn regulates signal transduction. The bacteria respond to the external environment through complex signal transduction pathways. Bacterial tyrosine kinases (BY-kinases) such as CapA-CapB were found to be involved in many important physiological functions such as stress response, antibiotic resistance, DNA metabolism, pathogenicity and cell cycle (Olivares-Illana et al., 2008; Chao et al., 2014). Tyrosine-protein kinase CapB (spot M36) was overexpressed in *P. pentosaceus* M41under all the stress treatments. This protein is involved in cell wall synthesis and could regulate growth of *P. pentosaceus* M41 under stress conditions. The conversion of free dihydroxyacetone (Dha) to Dha phosphate is mediated by Dha kinases in bacteria. Aldol cleavage of fructose-6-phosphate or glycerol oxidation is involved in the formation of Dha in bacteria (Siebold et al., 2003). Dihydroxyacetone kinase regulator (spot M37) was upregulated in *P. pentosaceus* M41 under heat, cold and bile stresses.

## Conclusion

Our study demonstrated that novel probiotic *P. pentosaceus* M41 showed tolerances against heat, cold, acid and bile stresses by overexpression of proteins involved in cellular defense and repair. The maximum number of differentially expressed proteins which were commonly expressed in all the stress treatments belonged to growth, repair, and energy metabolism such as proteins related to translation, carbohydrate metabolism, histidine biosynthesis, cell wall synthesis, DNA replication and stress response. The overexpression of these proteins could provide resistance to *P. pentosaceus* M41 against different environmental stresses. These proteins could be studied for their mechanism of action for enhancement of viability of *P. pentosaceus* M41 under different stress conditions and their potential use as probiotic markers. To the best of our knowledge this is the first study which demonstrates the proteomic response of *P. pentosaceus* M41 under different environmental stresses.

## Data Availability Statement

The raw data are available from Baig and Ayyash (2021).

## Author Contributions

MB: writing – original draft, investigation, data curation, and formal analysis. MA: conceptualization, design, writing – review and editing, supervising, and funding. AA-N and S-QL: writing – review and editing. MT and NS: conceptualization and writing – review and editing. All authors: contributed to the article and approved the submitted version.

## Conflict of Interest

The authors declare that the research was conducted in the absence of any commercial or financial relationships that could be construed as a potential conflict of interest.

## Publisher’s Note

All claims expressed in this article are solely those of the authors and do not necessarily represent those of their affiliated organizations, or those of the publisher, the editors and the reviewers. Any product that may be evaluated in this article, or claim that may be made by its manufacturer, is not guaranteed or endorsed by the publisher.
